# Kindergarten Obesity and Academic Achievement: The Mediating Role of Weight Bias

**DOI:** 10.3389/fpsyg.2021.640474

**Published:** 2021-04-16

**Authors:** Baeksan Yu

**Affiliations:** ^1^Norwegian Institute of Public Health (NIPH), Oslo, Norway; ^2^Faculty of Social Sciences, PROMENTA Research Center, University of Oslo, Oslo, Norway

**Keywords:** childhood obesity, teacher expectation, intersectionality, weight bias and stigma, weight-based discriminations

## Abstract

This study draws the attention towards the importance of reducing weight discrimination against children for their educational success, as an issue of social justice. We investigate the consequences of early-onset obesity identifying the mediating mechanisms in the relationship between childhood obesity and academic achievement. To do so, we employ the Early Childhood Longitudinal Study-Kindergarten Cohort (kindergarten to fifth grade) in the US (ECLS-K: 2011) and apply a parallel process latent growth model with a combination of quasi-experiments and econometrics. The results of this study suggest that teachers may serve as a significant source of weight bias, especially for girls (*B* = −0.09, 95% BC CI [−2.37 to −0.46]).

## Introduction

Early-onset obesity can be a significant predictor of student future academic success. Previous studies show that childhood obesity is significantly associated with an individual’s emotional distress/depression (Shaw et al., 2015), internalizing problem behaviors (Datar et al., 2004), resiliency (Shore et al., 2008), and with physical/cognitive impairments (Pacheco et al., 2017), which in turn affect educational outcomes (Caird et al., 2014). Importantly, the reported negative consequence of childhood obesity might stem from *weight bias* from peers, teachers, or even families (Puhl and Latner, 2007; Branigan, 2017). Previous studies, for instance, suggest negative effects of social marginalization and stigmatization of obesity by peers or teachers, especially for girls (Nutter et al., 2016; Martin et al., 2017) or minority groups (Crosnoe, 2007; Puhl et al., 2008).

Although teachers in general intend to be fair in their teaching practices, they sometimes set their expectations based on students’ previous academic performance, family backgrounds, sex, or race/ethnicity (Kelly, 2008; Kelly and Carbonaro, 2012). In particular, some studies suggest that teachers may perceive obese children to be overly emotional, disordered/untidy, or unattractive (Washington, 2011; Russell-Mayhew et al., 2015). That is, teachers can serve as a significant source of weight bias (Puhl and Latner, 2007). Indeed previous studies suggest that stigmatization or isolation from social interaction may function as a possible mediator between childhood obesity and academic performance (Caird et al., 2014). As children with obesity are already at higher risk for poor health outcomes (Smith et al., 2017), the weight-related discrimination/stigmatization in schools or other public areas has been widely criticized (Friedman, 2008). Some studies further argue that weight discrimination is comparable to the prevalence of racial discrimination in the US (e.g., Puhl et al., 2008), which would be striking given the pervasive concerns of racial bias in American society.

Despite the widespread belief, only few studies have empirically investigated mediating or moderating mechanisms between childhood obesity and educational outcomes [e.g., Gable et al., 2012; Kranjac, 2015, see also Santana et al. (2017)]. Importantly, from previous studies, it is difficult to disentangle whether observed negative effects of childhood obesity is due to obesity/overweight status *per se* or other unobserved child characteristics (e.g., previous cognitive and non-cognitive skills). When other things were functionally equal, would teachers’ negative evaluation of obese/overweight children be considered as weight bias. In addition, elementary school or teacher characteristics (e.g., school climate and teacher qualification) may also confound the relationship between teacher evaluation and student academic performance (i.e., mediator-outcome confounder). To address these likely confounding effects, we apply a parallel process latent growth model with a combination of quasi-experiments and econometrics.

Moreover, while several previous studies show how influences of childhood obesity differ by sex category, few studies have explored how the observed relationships differ at the *intersection* of sex and race/ethnicity [see also Branigan (2017), Puhl et al. (2008)]. Student identities are socially constructed in ways that are contingent upon context and culture; their identities are affected by how others perceive and evaluate them. For instance, the experiences of Black or Hispanic female students might be substantially different from other racial/ethnic groups (Cho et al., 2013), and the prevalence of obesity in childhood is more pronounced among many minority groups (Hales et al., 2017). Weight discrimination would be *double disadvantages* for minority students who are already at higher risk for poor health/psychological outcomes and are also exposed to other forms of discrimination.

This study seeks to contribute to previous studies by providing more robust empirical evidence on the longitudinal mediation process between kindergarten obesity, teacher evaluation, and academic achievement growth at the intersection of race/ethnicity, sex, and body size. It draws the attention toward the importance of reducing weight stigma and discrimination among children for their educational success, as an issue of social justice.

## Literature Review

### Childhood Obesity and Educational Outcomes

Why might being overweight or obese matter for student academic performance? A compelling explanation is that since obesity is generally associated with negative stigma or discrimination (e.g., claims of innuendo of being lazy, unintelligent, or dishonest), it may affect student self-esteem/efficacy or mental health, which in turn affects their academic outcomes (Puhl et al., 2008; Shaw et al., 2015; Harrist et al., 2016). Previous studies suggest that even young children are strongly biased against peers with obesity (Latner and Stunkard, 2003). Indeed, several studies show that being obese/overweight affects an individual’s emotional distress/depression (Shaw et al., 2015), internalizing problem behaviors (Datar et al., 2004), resiliency (Shore et al., 2008), or even levels of the stress hormone cortisol (Schvey et al., 2014). All of these outcomes could potentially serve as mediators between childhood obesity and academic achievement.

Second, the observed negative effects of childhood obesity on academic performance might be attributable to physical or cognitive impairments (Sabia, 2007). For instance, previous studies show that there is a significant association between obesity and sleep disorders (Sharma et al., 2017), and sleep disorders and academic achievement (Galland et al., 2015). Thus, there is a possibility that obesity may affect student academic performance via impaired physical function [see also Caird et al. (2014)]. Additionally, there might be direct effects of obesity on neurocognitive functioning, which affects cognition and behaviors. In particular, after reviewing articles from 1976 to 2013, Liang et al. (2014) conclude that there is a negative association between obesity and neurocognitive functioning such as attention or motor skills among adolescents. Cottrell et al. (2007) further suggest that increased cardiovascular risks among obese children may lead to lower academic performance.

Indeed, based on the ECLS-K data (1988 to 1999), Datar et al. (2004) show that overweight children tend to have lower math and reading test scores. The observed significant relationships might be explained by changes in child interpersonal skills and internalizing behaviors due to obesity (Gable et al., 2012). Yet, Crosnoe and Muller (2004) argue that the observed difference in GPA between obese and non-obese children is small. In the studies of LeBlanc et al., 2012 and Chen et al. (2012), they find no significant relationship between obesity and student academic achievement after controlling for family SES. In this regard, Santana et al. (2017) conclude that there is no compelling evidence for the significant direct impacts of obesity on academic performance among school age children; they suggest that more rigorous longitudinal research is needed.

Importantly, related to the analyses in this study, few studies have empirically investigated possible mediating mechanisms between childhood obesity and educational outcomes formally, even as the many persuasive mechanisms discussed above have been advanced [see Puhl and Latner (2007), Puhl and Heuer (2009), Santana et al. (2017)]. The lack of formal testing might be accounted for by a tendency among many social science researchers to rely first on simple “X –> Y tests” in determining a necessity of mediational analyses. However, a null effect of obesity on academic performance does *not* necessarily mean that there are no mediation effects. Since the total effect of X on Y is the sum of the direct and indirect effects, as an example, opposite signs may cancel each other out [see more discussion in Hayes (2009) or Zhao et al. (2010)].

### Teachers and Weight Bias

Weight bias in general refers to negative attitudes toward individuals because of obesity or overweight status (Puhl et al., 2014). There is growing evidence that stigmatization and discrimination toward overweight and obese children may be a major social problem (Puhl and Heuer, 2009). Although teachers in general intend to be fair in their teaching practices, and they are trained and socialized to be fair (Valenzuela, 2016), teachers often set their expectations based on students’ previous academic performance, family SES, or race/ethnicity and sex congruence (Tenenbaum and Ruck, 2007; McKown and Weinstein, 2008). Teachers, for instance, often perceive low-track students as more inattentive, disruptive, and withdrawn and place an excessive emphasis on discipline (Kelly and Carbonaro, 2012). In contrast, teachers tend to provide more feedback, praise, and challenging instruction for high-expectation students (Cooper, 1979; Rubie-Davies, 2007). Importantly, even young children are able to identify teachers with different expectations (Peterson et al., 2016), and how teachers perceive students affects student academic performance via the many teacher-student interactions in daily class (Rubie-Davies, 2007; Hattie, 2009; Rubie-Davies et al., 2015). For instance, Rubie-Davies et al. (2015), based on an RCT, show that students in classrooms of teachers with high expectations tend to have higher math scores.

Previous studies also suggest that weight bias among educators may affect obese or overweight students’ academic performance, though empirical evidence is limited (Puhl and Latner, 2007; Caird et al., 2014). In particular, specific studies have found that teachers are likely to have lower expectations for obese/overweight children (Friedman, 2008), and they also perceive obese children as being emotional, unmotivated, less competent, and non-compliant (Washington, 2011; Puhl and Peterson, 2012; Russell-Mayhew et al., 2015). Mahoney et al. (2005), for instance, show that even after accounting for differences in poverty status and race/ethnicity, teacher-rated popularity for children is significantly lower for obese children. Obese students are also found to experience discrimination or stigmatization from their teachers (e.g., Puhl and Brownell, 2006; Finn et al., 2020; Dian and Triventi, 2021).

What underlying mechanisms might potentially explain weight discrimination or stigmatization among teachers? According to *attribution theory*, individuals tend to seek causes and make attributions (i.e., specific attributional tendencies of blame), when they encounter a person with stigmatized characteristics (Puhl and Peterson, 2012). A prevailing societal perception in the US is that since BMI is modifiable, obese people are to blame for being overweight (e.g., low self-discipline or impulsivity). The perception may be further strengthened by US cultural beliefs that emphasize meritocratic values [for more discussion of meritocracy see Bills (2019)]. Moreover, individuals are often exposed to gendered and racialized cultural stereotypes about their physical appearance by schooling, media, and their families (e.g., Western ideals of thinness and beauty). Previous studies, for instance, show that females (Barry and Grilo, 2002) or White females (Wang et al., 2009) are more concerned about eating and body image disturbances.

Indeed, previous studies suggest that the negative effects of stigmatization and discrimination of obesity by peers or teachers might be more salient for girls (Tang-Péronard and Heitmann, 2008; Martin et al., 2017). For instance, Datar and Sturm (2006) find that the significant association between overweight status and school outcomes (e.g., test scores or approaches to learning) does not hold for boys. Regarding racial/ethnic groups, Puhl et al. (2008) suggest that weight discrimination might be more prevalent in minorities such as Black girls. Crosnoe (2007) also points that the association between obesity and college enrollment is stronger for girls from racial/ethnic minority groups. Yet, the empirical studies on marginalized subpopulation who experiences multiple discrimination (e.g., ethnic/racial minorities or LGBTQ individuals) are still lacking.

### Research Questions

Consequently, the main research questions of this study are: (1) Are there any mediating effects of teacher evaluation on obese children’s academic achievement? (2) If so, do mediation effects differ by sex or minority girls? The findings of this study will provide empirical evidence on the links between kindergarten obesity, teacher evaluation, and academic achievement and offer critical information about the influence of weight discrimination and stigmatization.

## Methods

### Data and Sample

To achieve the aim of this study, we employ the newly released Early Childhood Longitudinal Study-Kindergarten Cohort (ECLS-K: 2011), which is a nationally representative sample of American children who entered kindergarten in 2010–2011. The ECLS-K study follows the kindergarten cohort of 2010–2011 through the 2015–2016 school year, providing a comprehensive picture of children’s academic development until secondary school. The study also includes a wide range of data on the children, their homes, and school environments based on a three-step sampling design [for more information on the ECLS-K, see Tourangeau et al. (2015)]. Approximately 18,170 kindergarteners from 1,310 schools were sampled in the baseline year. This study employs the data from kindergarten to fifth grade. The final analytic sample is 15,820. Sample sizes are rounded to the nearest 10 in accordance with NCES secure data.

### Measures

#### Obesity and Overweight Status

We create BMI-based obesity and overweight specifications at kindergarten based on the composite BMI calculated by composite weight and height in the ECLS-K (Hsu et al., 2019). To obtain accurate measurements, each child’s height and weight were measured twice in each data collection using a Shorr board and a digital scale. Composite BMI was then computed based on the composite height and weight measures, which were constructed from two measurements [see more in Tourangeau et al. (2015)]. Overweight children are defined as being between the 85th and 95th percentiles of BMI, while obese children are above the 95th percentile of BMI (Staiano et al., 2013; DeFrancesco et al., 2018)^1^.

#### Non-cognitive Skills

The ECLS-K provides a set of reliable measures of children’s non-cognitive skills widely used in previous studies (e.g., Datar and Sturm, 2006; Liu, 2019). We use composite variables representing teacher perception of children’s social skills and behavioral problems provided in the ECLS-K. Teachers reported how often their children exhibited certain social skills and behavioral problems using a scale ranging from “never” to “very often.” These are teacher report of approaches to learning (e.g., eagerness to learn new things), self-control (e.g., controlling temper or accepting peer ideas), interpersonal skills (e.g., skills in forming and maintaining friendships), and externalizing (e.g., whether a child argues, fights, gets angry, acts impulsively, reverse coded) and internalizing problem behaviors (e.g., presence of anxiety, loneliness, low self-esteem, and sadness, reverse coded). Higher scores indicate that the child shows the behavior represented by the scale more often. Cronbach’s alpha computed from the five items is 0.88.

#### Academic Achievement

We use the reading and math IRT scores widely used in previous studies (e.g., Kranjac, 2015; Little, 2017). IRT scoring makes possible longitudinal measurement of gain in achievement, even though the assessments administered to a child are not identical [see more in Tourangeau et al. (2015)]. We use both reading and math IRT scores that were measured from grade 1 to 5 (Little, 2017).

#### Confounders

Based on previous studies (O’Malley et al., 2007; Murasko, 2009), this study identifies student, family, and school-level potential confounders measured at kindergarten. The aim of matching is to reduce as much of the difference between treatment and control (Lee, 2016). We thus include a variety of potential confounders measured at kindergarten in generating CBPS weights (see more in the analytic section). These include child sex, age, race/ethnicity, birth weight, attendance of pre-kindergarten programs (e.g., pre-school or Head Start), parental reports of overall child health, child’s disability status, family income, family size, single parent, parent’s educational level and educational expectations for children, home language (English or not), participation in cultural activities (e.g., visiting museums or theaters), children’s math, science, reading IRT scores, residential area, school locale, school type (private vs. public), school SES, school size, and percentage of Black or Hispanic children at schools. The descriptive statistics of the variables are available in Appendix B.

### Analytic Strategy

**Figure 1:**
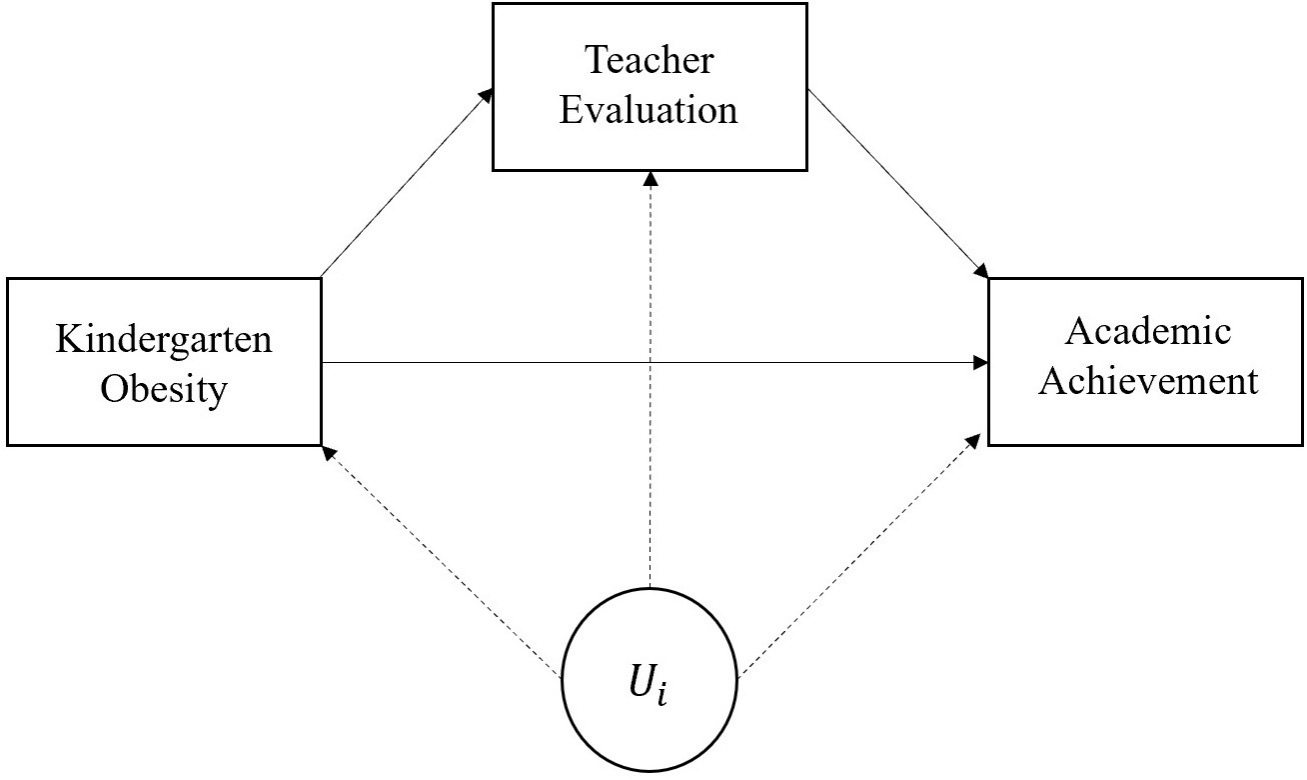
Baseline mediation model and unobserved heterogeneity.

The current study investigates the longitudinal mediation process between kindergarten obesity, teacher evaluation, and academic achievement among marginalized subpopulations. Figure 1 illustrates the proposed mediation process with potential unobserved heterogeneity. From previous correlational cross-sectional studies, it is unclear whether teachers’ perceptions of obese students’ non-cognitive skills are biased or not, due to systematic initial differences in children, families, and school characteristics other than obesity status *per se* (*U*_*i*_ in Figure 1); we first need to block the backdoor from kindergarten obesity to *U*_*i*_ to obtain unbiased direct effects of kindergarten obesity on teacher evaluation and academic achievement. It is also questionable, even if childhood obesity may lead to teachers’ negative perceptions, whether the teachers’ negative evaluation can serve as a significant mediator between childhood obesity and academic performance; unobservable differences among teachers or schools may account for the observed relationships (effects of *U*_*i*_ both on teacher evaluation and academic achievement in Figure 1); the mediator-outcome confounding needs to be addressed to obtain unbiased indirect effects of kindergarten obesity on academic achievement.

To address unobserved heterogeneity among obese and overweight children (i.e., to block the backdoor from kindergarten obesity to *U*_*i*_ in Figure 1), we first calculate covariate balancing generalized propensity scores for subsequent analytic models. The key feature of the method is that it can be applied to a continuous or categorical treatment variable with the improvement of the robustness to model misspecification in matching and weighting by optimizing sample covariate balance [see more in Fong et al. (2018)]. Based on the potential confounders (e.g., birth weight, previous academic achievement, or family/school SES) that were measured at kindergarten, we first set the treatment assignment [being overweight (=1) and obese (=2)] equation and calculate CBPS weights. For subgroup analyses, CBPS weights are calculated within each subgroup. The estimated CBPS weights can be employed in the standard regression setting. The covariate balance with CBPS weights between treatment groups is illustrated in Appendix A; covariate balances between treatment groups in this analysis are excellent in terms of standardized mean differences. To account for possible mediator-outcome confounding, we then apply teacher- and school-fixed effects models as well as random-intercept latent variable approach (Muthén and Asparouhov, 2019); the fixed-effects approach can effectively remove systematic stable differences in teacher and school characteristics.

**Figure 2:**
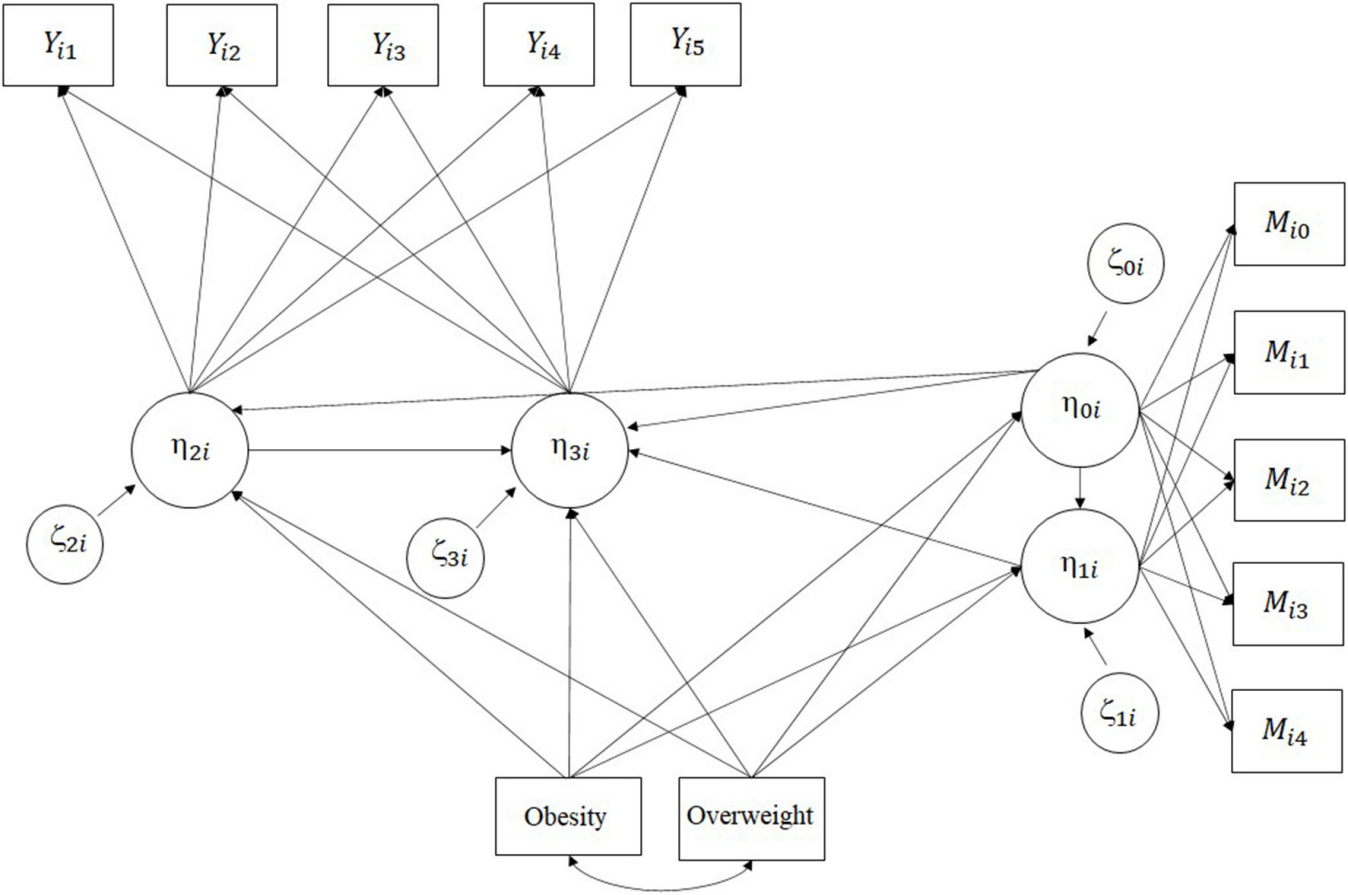
PP-LGM for mediation analysis.

Based on the identification strategy, we then apply a parallel process latent growth model (PP-LGM). When both the dependent variable and mediating variable are measured repeatedly over time, the growths of dependent and mediating variables can be considered as two distinctive LGM processes (Cheong et al., 2003). Figure 2 illustrates a parallel process growth curve model (or bivariate latent growth model) where children’s academic achievements and non-cognitive skills are measured over five time points with a linear specification. Note that while the LGM for student academic achievement is measured from grade 1 to 5 (intercept is grade 2), the LGM for teacher report of non-cognitive skills is measured from kindergarten to 4th grade (intercept is grade 1). The time specification is necessary to make an appropriate time sequence in the mediation process.

The proposed mediation model can be written as follows:

(1) $$\begin{array}{ll} \eta_{2i} & =\eta_{2}+\gamma_{21}\left(obesity/overweigth\right)+{\gamma_{22}\eta }_{0i}+\zeta_{2i} \end{array}$$

(2) $$\begin{array}{l} \eta_{3i}=\eta_{3}+\gamma_{31}\left(obesity/overweigth\right)+\gamma_{32}\eta_{0i}+\gamma_{33}\eta_{1i}\\ \;+\gamma_{34}\eta_{2i}+\zeta_{3i} \end{array}$$

(3) $$\begin{array}{rc} \eta_{0i} & =\eta_{0}+\gamma_{01}\left(obesity/overweight\right)+\zeta_{0i} \end{array}$$

(4) $$\begin{array}{rc} \eta_{1i} & =\eta_{1}+\gamma_{11}\left(obesity/overweigth\right)+{\gamma_{12}\eta }_{0i}+\zeta_{1i} \end{array}$$

where *η*_0*i*_ represents the initial status for the mediation process (i.e., teacher report of non-cognitive outcomes); *η*_1*i*_ is the growth rate of the mediation process; *η*_2*i*_ is the initial status for the outcome variable (i.e., child academic achievement); *η*_3*i*_ is the growth rate of the outcome variable. While the growth rate of the outcome in equation (2) is predicated by both the initial status (*η*_0*i*_) and growth (*η*_1*i*_) of the mediation process, the initial status of the outcome (*η*_2*i*_) is only predicated by the initial status of the mediation process (*η*_0*i*_). In both LGMs, latent growth factors are predicted by latent intercepts to account for their associations (Von Soest and Hagtvet, 2011). Note also that each latent factor, *η*_*ki*_, is predicated by our focal variables, kindergarten obesity and overweight status (two rectangles in the bottom of Figure 2).

In the proposed PP-LGM, we are particularly interested in the indirect effects of teacher evaluation between kindergarten obesity/overweight status and academic achievement growth. Since a higher order time specification for academic achievement growth yields a model convergence issue, we use a latent basis model for academic growth by freely estimating growth parameters (Grimm et al., 2011), which also exhibits excellent model fit indices (see more in the results section). Mediation analyses are conducted with a bias-corrected bootstrapping (Preacher and Hayes, 2008) with 2,000 replications. Given the nested structure of the ECLS-K data, cluster option is employed to adjust standard errors with maximum likelihood with robust standard errors (MLR or Huber–White SEs) which is robust to non-normality. Missing cases are imputed with a multiple imputation generating 10 data sets. R and Mplus are employed to conduct the proposed methods.

## Results

### Mediating Roles of Teachers’ Evaluation of Students’ Non-cognitive Skills

Before investigating indirect effects of teacher’s evaluation of students’ non-cognitive skills, we first explore appropriate time specifications for each LGM part of the PP-LGM in Figure 2. We consider linear, quadratic, cubic, and latent basis models. It is recommended that CFI and TLI should be higher than 0.90 and RMSEA should be lower than 0.08 (Hooper et al., 2008). Based on the model fit indices, we select a latent basis model for math and reading scores and a linear model for teacher report of students’ non-cognitive skills; the combined model fits are excellent in terms of RMSEA, CFI, and TLI, implying that the proposed models are reasonably consistent with the ECLS-K data (see Tables 1–**3**). All mediation analyses are adjusted with CBPS weights to account for unobserved heterogeneity among children with obesity.

**Table 1:** PP-LGM mediation model for total students (*n* = 15,820).

**Model fit**	**RMSEA**	**CFI**	**TLI**		**Indirect effects**	
	**0.02**	**0.99**	**0.99**	**b**	**B**	**BC bootstrap 95% CI**
**Mediation process (reading)**						
Overweight (K) (–)->Non-cog skills intercept (1st) (+)->Reading intercept (2nd)				−0.68^**^ (0.21)	−0.05^**^ (0.01)	[−1.07, −0.29]
Obesity (K) (–)->Non-cog skills intercept (1st) (+)->Reading intercept (2nd)				−0.71^*^ (0.34)	−0.05^*^ (0.02)	[−1.49, −0.21]
**Model fit**	**RMSEA**	**CFI**	**TLI**		**Indirect effects**	
	**0.02**	**0.98**	**0.98**	**b**	**B**	**BC bootstrap 95% CI**
**Mediation process (math)**						
Overweight (K) (–)->Non-cog skills intercept (1st) (+)->Math intercept (2nd)				−0.65^**^ (0.20)	−0.04^**^ (0.01)	[−1.01, −0.27]
Obesity (K) (–)->Non-cog skills intercept (1st) (+)->Math intercept (2nd)				−0.68^*^ (0.33)	−0.04^*^ (0.02)	[−1.42, −0.20]

* *p < 0.05*,

** *p < 0.01*,

^***^p < 0.001.

*Time specification is based on a latent basis model. Standard errors are in parentheses. The significant p-values of unstandardized and standardized coefficients are obtained from MLR*.

**Table 2:** PP-LGM mediation model for boys (n = 8,070).

**Model fit**	**RMSEA**	**CFI**	**TLI**		**Indirect effects**	
	**0.02**	**0.99**	**0.98**	**b**	**B**	**BC bootstrap 95% CI**
**Mediation process (reading)**						
Overweight (K) (–)->Non-cog skills intercept (1st) (+)->Reading intercept (2nd)				−0.62^*^ (0.28)	−0.02^*^ (0.01)	[−1.11, −0.05]
**Model fit**	**RMSEA**	**CFI**	**TLI**		**Indirect effects**	
	**0.02**	**0.99**	**0.98**	**b**	**B**	**BC bootstrap 95% CI**
**Mediation process (math)**						
Overweight (K) (–)->Non-cog skills intercept (1st) (+)->Math intercept (2nd)				−0.71^*^ (0.32)	−0.04^*^ (0.02)	[−1.37, −0.15]

* *p < 0.05*,

^**^p < 0.01,

^***^p < 0.001.

*Time specification is based on a latent basis model. Standard errors are in parentheses. The significant p-values of unstandardized and standardized coefficients are obtained from MLR*.

**Table 3:** PP-LGM mediation model for girls (*n* = 7,730).

**Model fit**	**RMSEA**	**CFI**	**TLI**		**Indirect effects**	
	**0.02**	**0.99**	**0.99**	**b**	**B**	**BC bootstrap 95% CI**
**Mediation process (reading)**						
Overweight (K) (–)->Non-cog skills intercept (1st) (+)->Reading intercept (2nd)				−0.95^**^ (0.34)	−0.07^**^ (0.02)	[−1.58, −0.25]
Obesity (K) (–)->Non-cog skills intercept (1st) (+)->Reading intercept (2nd)				−1.21^*^ (0.52)	−0.09^*^ (0.04)	[−2.37, −0.46]
**Model fit**	**RMSEA**	**CFI**	**TLI**		**Indirect effects**	
	**0.02**	**0.98**	**0.98**	**b**	**B**	**BC bootstrap 95% CI**
**Mediation process (math)**						
Overweight (K) (–)->Non-cog skills intercept (1st) (+)->Math intercept (2nd)				−0.96^**^ (0.35)	−0.07^**^ (0.03)	[−1.68, −0.29]
Obesity (K) (–)->Non-cog skills intercept (1st) (+)->Math intercept (2nd)				−1.23^*^ (0.52)	−0.09^*^ (0.04)	[−2.41, −0.47]

* *p < 0.05*,

** *p < 0.01*,

^***^p < 0.001.

*Time specification is based on a latent basis model. Standard errors are in parentheses. The significant p-values of unstandardized and standardized coefficients are obtained from MLR*.

The results of PP-LGM are illustrated in Tables 1–**3** with bias-corrected (BC) bootstrap 95% confidence intervals (Preacher and Hayes, 2008). We report both the results from MLR and ML with BC bootstrapping, which are robust to violation of multivariate normality; they provide generally equivalent results (Yuan and Hayashi, 2006). Teacher reports of multiple non-cognitive skills are averaged to represent an overall level of children’s non-cognitive skills^2^.

Table 1 (for total students) shows that kindergarten obesity/overweight status affect students’ reading and math achievement intercepts via teacher evaluation. Specifically, kindergarten obesity/overweight status are related to teachers’ negative evaluation of students’ non-cognitive skills at grade 1, which in turn affect the intercepts of reading/math scores (at grade 2). There are no significant indirect effects via growth factors. Yet, the estimated standardized coefficients are small in the model for all students (0.04–0.05, *p* < *0.05*).

Previous studies report that negative effects of stigmatization of obesity might be more salient for girls (Tang-Péronard and Heitmann, 2008; Martin et al., 2017). We thus further investigate how the observed mediation effects differ between boys and girls, and illustrate findings in Tables 2, 3. Notably, observed mediation effects are also pronounced for girls, and obesity status is more predictive than overweight status for girls. In particular, for girls, reading/math scores at grade 2 decrease by 0.09 standard deviations (*p* < *0.05*) for being obese via teacher evaluation (see Table 3). We observe the similar pattern both for reading and math subjects in this study. Given the pooled effect size between perceived racial/ethnic discrimination experiences and academic outcomes among adolescents is about 0.10 (Benner et al., 2018), the observed mediation effects seem non-trivial.

Given that weight stigmatization/discrimination is hypothesized to be more pronounced for minority groups (Puhl et al., 2008), we further explore whether the mediation effects of teachers’ evaluation are particularly harmful for Black and Hispanic girls. We observe that, for Hispanic girls, reading/math scores at grade 2 decrease by 0.14 standard deviations for being obese via teacher evaluation at *p* < *0.10*. The estimated standardized coefficient for Black girls is also sizeable (*B* = −0.19), though it is not statistically significant. Thus, emphasis should be placed on the results from girls.

### Robustness Check

#### Disentangling Weight Bias

Weight bias refers to discriminatory or prejudicial attitudes toward individuals because of an individual’s bodyweight itself. When other things were functionally equal, would teachers’ negative evaluation of obese children be considered as actual weight bias. It is thus important to address heterogeneity among obese/overweight children (e.g., previous academic performance, health conditions, and family/school SES) to accurately evaluate consequences of weight discrimination/stigmatization among obese children. This study accounts for the observed initial differences between obese and non-obese kindergarteners using well-balanced CBPS weights. Yet, there is a possibility that kindergarten obesity/overweight status may affect teacher evaluation via concomitant changes in self-esteem/efficacy or difficulties in school adaptation among obese children (e.g., peer relationships). That is, the observed significant negative effects might be the sum of weight bias along with reactions to the potential detrimental effects of kindergarten obesity on students’ non-cognitive skills. Given that the observed significant effects of kindergarten obesity are mainly limited to intercepts (Spring 1st grade), however, it is less likely that children’s social skills or problematic behaviors meaningfully change within a half semester during 1st grade due to unobserved factors. Yet, one possible way to disentangle weight bias from other sources is to further control for parent reports of children’s social skills: self-control; social interactions; sad/lonely designations; impulsive/overactive designations that are available at grade 1. We examine how the estimated coefficients for girls change after controlling for parent reports of social skills (see second column of Table 4). The results are very similar suggesting that the observed negative teacher evaluation of obese girls might be accurately termed as weight bias.

**Table 4:** Sensitivity analysis for mediational analyses.

**Mediation paths**	**PP-LGM**	**Parent report controlled**	**School fixed**	**Latent U_i_**
**Total**				
Overweight-> Non-cog skills intercept-> Reading intercept	−0.68^**^ (0.21)	−0.63^**^ (0.19)	−0.66^**^ (0.24)	−0.67^**^ (0.23)
Obesity-> Non-cog skills intercept-> Reading intercept	−0.71^*^ (0.34)	−0.93^**^ (0.32)	−0.80^*^ (0.36)	−0.70^*^ (0.33)
Overweight-> Non-cog skills intercept-> Math intercept	−0.65^**^ (0.20)	−0.62^**^ (0.19)	−0.64^**^ (0.23)	−0.65^**^ (0.20)
Obesity-> Non-cog skills intercept-> Math intercept	−0.68^*^ (0.33)	−0.91^**^ (0.31)	−0.77^*^ (0.35)	−0.67^*^ (0.33)
**Boys**				
Overweight-> Non-cog skills intercept-> Reading intercept	−0.62^*^ (0.28)	−0.63^*^ (0.26)	−0.49 (0.31)	−0.63^*^ (0.28)
Overweight-> Non-cog skills intercept-> Math intercept	−0.71^*^ (0.32)	−0.72^*^ (0.29)	−0.56 (0.35)	−0.68^*^ (0.31)
**Girls**				
Overweight-> Non-cog skills intercept-> Reading intercept	−0.95^**^ (0.34)	−0.77^*^ (0.30)	−1.01^**^ (0.38)	−0.93^**^ (0.32)
Obesity-> Non-cog skills intercept-> Reading intercept	−1.21^*^ (0.52)	−1.34^**^ (0.49)	−2.11^**^ (0.67)	−1.19^*^ (0.53)
Overweight-> Non-cog skills intercept-> Math intercept	−0.96^**^ (0.35)	−0.81^*^ (0.31)	−1.04^**^ (0.39)	−0.93^**^ (0.34)
Obesity-> Non-cog skills intercept-> Math intercept	−1.23^*^ (0.52)	−1.40^**^ (0.50)	−2.15^**^ (0.68)	−1.22^*^ (0.52)

* *p < 0.05*,

** *p < 0.01*,

^***^p < 0.001.

#### Omitted Variable Bias

Although this study employs well-balanced CBPS weights between treatment groups using multiple covariates, it is worthwhile to reiterate that matching/weighting methods are based on the conditional independence assumption (Morgan and Winship, 2007); there might also be unobserved time-varying confounding. Yet, since the observed significant effects are mainly limited to intercepts of models, the estimated coefficients are likely to be robust to unobserved time-varying confounding. As a supplemental analysis, we further control for observed time-varying covariates such as family income and family structure; the main results are also very similar. In addition, in this longitudinal mediation model, elementary school or teacher characteristics (e.g., school climate, school SES, and teacher qualification) may also confound the relationship between teacher evaluation and student academic performance (i.e., mediator-outcome confounder) (Keele, 2015). To address this concern, we apply school fixed-effects models in the PP-LGM framework^3^. Additionally, we also generate a phantom latent unobserved heterogeneity [or random intercept see Muthén and Asparouhov (2019)] that has constant effects on mediator and outcome variables (Finkel, 2008). The results are illustrated in Table 4. The school fixed-effects and latent unobserved heterogeneity approaches show broadly similar findings, providing confidence in the mediation results, especially for girls.

## Discussion

In the present study, we investigated the consequences of early-onset obesity identifying the mediating mechanisms in the relationship between childhood obesity and academic achievement. In the US, weight discrimination has increased by 66% over the past decade and is also comparable to the prevalence of racial discrimination, especially for females or minority groups (Puhl et al., 2008; Puhl and Heuer, 2009). Hebl et al. (2019) also identify weight discrimination as one of a few distinctly modern forms of discrimination against e.g., LGBTQ individuals and older adults.

Importantly, children are vulnerable to weight discrimination and stigmatization (Puhl and Peterson, 2012), and how teachers perceive students affects student academic performance via teacher-student interactions in daily class (Kelly and Carbonaro, 2012; Rubie-Davies et al., 2015). Compared to the general public reaction to racial and gender discrimination in the US, however, weight-related discrimination has been often rationalized and justified in many public areas. A recent news article, for instance, highlights that although female overweight candidates are more likely to be judged harshly in a job market, federal anti-discrimination laws provide little or no protection for overweight employees (Martin, 2017).

Despite the widespread belief, however, few empirical studies have directly investigated whether and how teacher evaluation on obese children matters for student academic achievement in elementary schooling. Importantly, previous studies are limited to cross-sectional correlational studies (Santana et al., 2017) and have paid little attention to the intersection of race/ethnicity, sex, and body size. This study is among the first to investigate the longitudinal mediation process between kindergarten obesity, teacher evaluation, and academic achievement growth among marginalized subpopulations with a more rigorous research design.

The results of this study demonstrated that there are significant mediating effects of teacher evaluation of obese/overweight children’s non-cognitive skills, which is also consistent with the previous finding that teacher report of interpersonal skills/internalizing behaviors might matter for children’s math performance (Gable et al., 2012). In particular, the mediation effects were more pronounced for girls; reading/math scores decreased by −0.09 standard deviations for kindergarten obesity via teacher evaluation, which were also robust to multiple specifications including teacher and school fixed-effects models. Given the pooled effect size between perceived racial/ethnic discrimination experiences and academic outcomes among adolescents, from a meta-analysis, is about 0.10 (Benner et al., 2018), the observed mediation effects suggest that negative influence of weight stigmatization or discrimination might be comparable to racial/ethnic discrimination. We also observed that the mediation effect is particularly substantial for Hispanic girls (*B* = −0.14, *p* < *0.10*), though it is marginally significant.

Why is the observed negative mediating effect more pronounced for girls? Our finding is consistent with previous report that negative effects of stigmatization/discrimination of obesity might be more salient for girls (Tang-Péronard and Heitmann, 2008; Martin et al., 2017). Perhaps, a compelling explanation is that the body images or gendered cultural stereotype about girls’ physical appearance [e.g., women with obesity are judged unfeminine or unattractive, see also Pine (2001), Sabia and Rees (2015)] make girls more exposed to negative rates by peers or teachers. Branigan (2017), for instance, shows that the negative association between obesity and teacher-assessed academic achievement is larger for White girls in English, which is a traditionally female-gendered subject. Our finding thus highlights the importance of intersection of sex and body size in investigating heterogeneous effects of obesity.

As a whole, the results of this study suggest that teachers may serve as a significant source of weight bias, which in turn affects child academic performance. Although the observed negative mediation effects were not cumulative (i.e., no growth effects) and relatively small, it can be an additional disadvantage for minority students who are already at higher risk for poor health/psychological outcomes and are also exposed to other forms of discrimination. Importantly, since kindergarten obesity/overweight status are closely related to family background, kindergarten weight problems would be “double jeopardy” for minority students due to the lack of family resources.

### Policy Implications

From a policy perspective, this study draws the attention toward the importance of reducing weight stigma and discrimination among children for their educational success, as an issue of social justice. It suggests the need to incorporate weight and health education into teacher professional development, so that teachers can serve as a preventive actor in reducing the detrimental effects of kindergarten weight problems; these include emphasizing health and quality of life, stop disseminating curriculum materials that has negative weight bias, and creating inclusive physical activities encompassing students with high body weight [see more in Russell-Mayhew et al. (2015); Ramos Salas et al. (2017)]; Rudd Center for Food Policy & Obesity provides more resources for schools/educators. They may need to be aware of that the negative effects of weight discrimination/stigmatization may be comparable to racial discrimination, as this study suggests. It should be, however, noted that teachers alone cannot solve the weight discrimination. Fostering a positive and supportive school climate, for instance, may also help obese children to avoid weight discrimination/stigmatization (Winter, 2009; Fair et al., 2018). More importantly, since major risk factors for child obesity lie outside of schools (Von Hippel and Workman, 2016), careful monitoring of children with early-onset obesity should also take place within families and schools with obesity intervention programs [see also Bleich et al. (2017), Jakicic and Davis (2011)]. Replacing the widespread societal stereotypes of obesity, with greater tolerance for diverse body types and physical characteristics (Walker, 2014), would also be necessary.

### Limitations and Future Studies

This study explores heterogeneous effects of kindergarten obesity/overweight status on children’s cognitive and non-cognitive outcomes among marginalized girls who are at the intersection of race/ethnicity, sex, and boy size. Yet, it may be that the observed relationships can further vary by teacher or school characteristics. For instance, previous studies on teacher-student race/sex matching suggest that teachers may have different expectations based on the racial or sex congruence (e.g., Weathers, 2019), though Pigott and Cowen (2000) find no significant interactions between teacher and student race/ethnicity. In addition, in schools where obesity is not the norm (e.g., high-SES private school), obese/overweight children may face multiple disadvantages [see also Crosnoe and Muller (2004)]. In this regard, Benner and Wang (2015) suggest the importance of the influence of school demographics. Yet, careful attention should be paid to the establishment of causality in multiple treatment variables in complex interaction models [see more in VanderWeele (2015)]. It is also worthwhile to mention that there are various obesity indexes other than BMI-based obesity classification such as waist circumference or waist hip ratio [see also Bener et al. (2013)]; unfortunately, these information are limited in the ECLS-K. Finally, although this study attempts to account for unobserved heterogeneity by applying quasi-experiments, there might be unobserved time-varying confounding related to specific genetic or neurotropic factors found in obese children [see also Mora-Gonzalez et al. (2019)]; the possibility of exposure-mediator interaction is not also fully considered in this study (VanderWeele and Ding, 2017). Expanding the analytic model of this study to include heterogeneity of teacher and school characteristics with more rigorous methods will enrich our current understanding of the psychological process between childhood obesity and educational attainments.

## Data Availability Statement

The datasets presented in this study can be found in online repositories. The names of the repository/repositories and accession number(s) can be found at: https://nces.ed.gov/ecls/.

## Ethics Statement

The study employed Early Childhood Longitudinal Study-Kindergarten Cohort (ECLS-K: 2011), which is a nationally representative sample of American children who entered kindergarten in 2010-2011. All students gave their informed consent prior to their participation in the study. This study was reviewed and approved by IES Data Security.

## Author Contributions

The author confirms being the sole contributor of this work and has approved it for publication.

## Conflict of Interest

The author declares that the research was conducted in the absence of any commercial or financial relationships that could be construed as a potential conflict of interest.

## Footnotes

^1^It is worthwhile to mention that there are different ways to measure obesity/overweight status (e.g., SD of BMI or total body fat), and there are also controversies over the BMI-based classification [see Karchynskaya et al. (2020)].

^2^Unfortunately, a latent variable approach is not feasible due to the model complexity of PP-LGM with CBPS weight. The overall Cronbach’s alpha computed from the 5 items is 0.88, and the standardized factor loadings of the items in CFA are >0.8.

^3^The results from teacher fixed-effects model are not reported here. Because of the small number of students in each teacher compared to the school level, the within-group estimates may not be reliable (Cameron and Trivedi, 2009). Yet, we still observed significant mediation effects for girls at *p* < *0.05*.
