# Supplementary material for: Kindergarten Obesity and Academic Achievement: The Mediating Role of Weight Bias
Source: Front Psychol. 2021 Apr 16;12:640474. doi: 10.3389/fpsyg.2021.640474 (PMC8086407; doi:10.3389/fpsyg.2021.640474)
Supplement: Supplementary file 1 [file Data_Sheet_1.doc]

**Appendix A**. Covariate balance between treatment groups (1=ref, 2=overweight, 3=obesity)


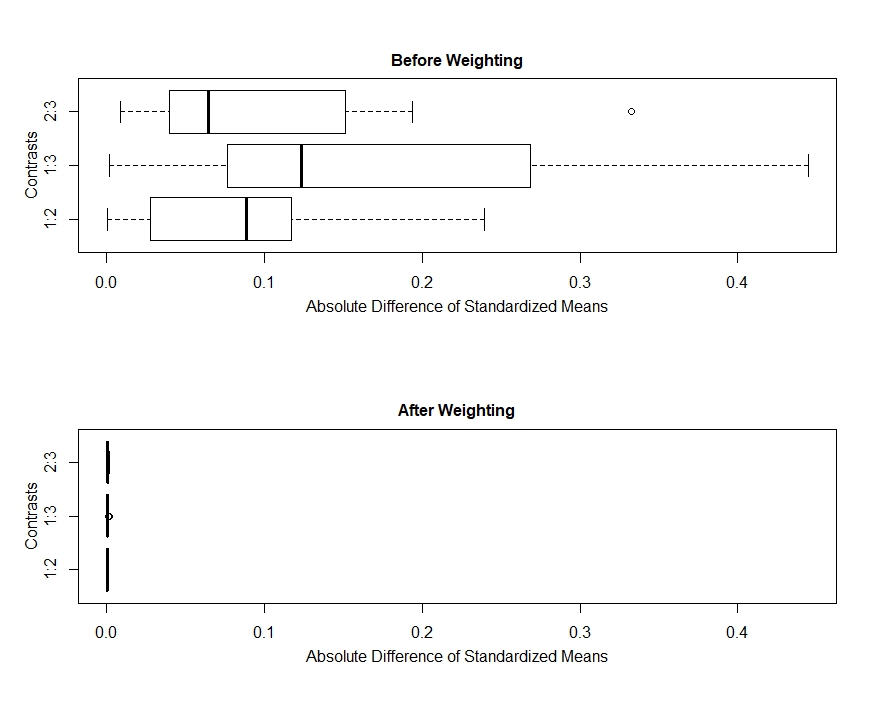

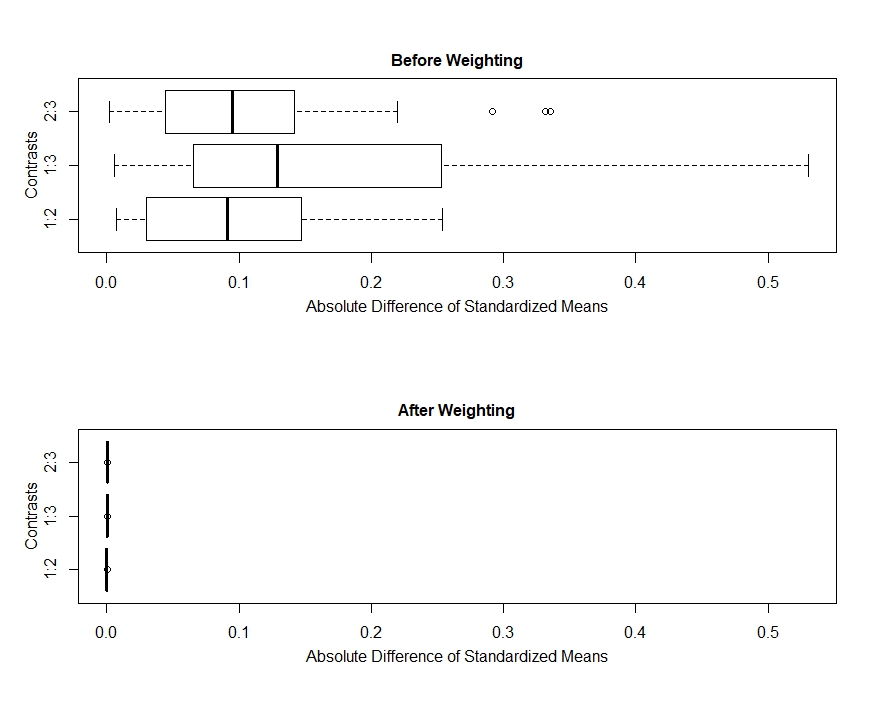


Total Male


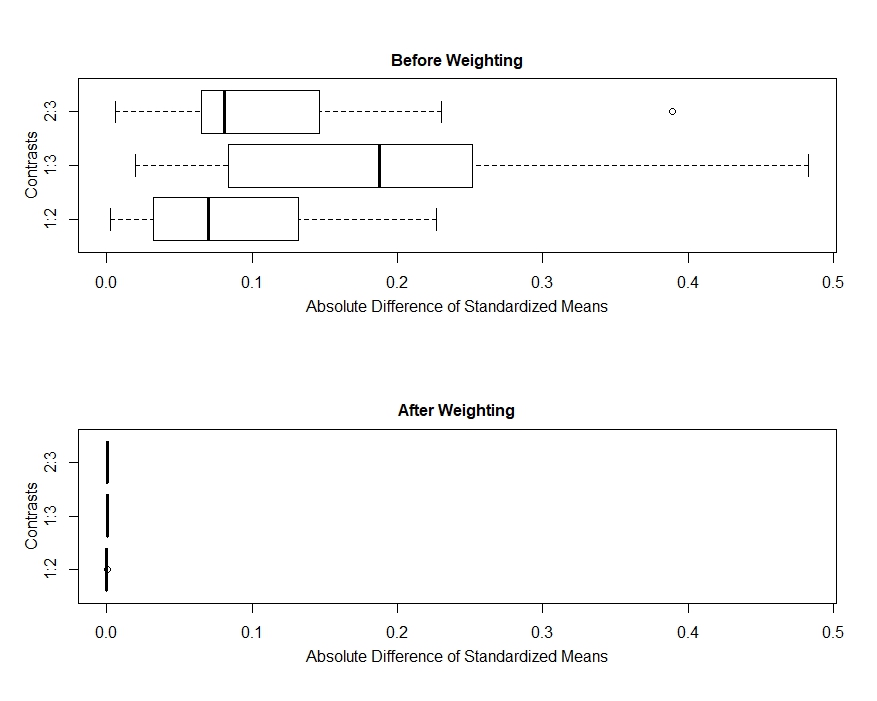

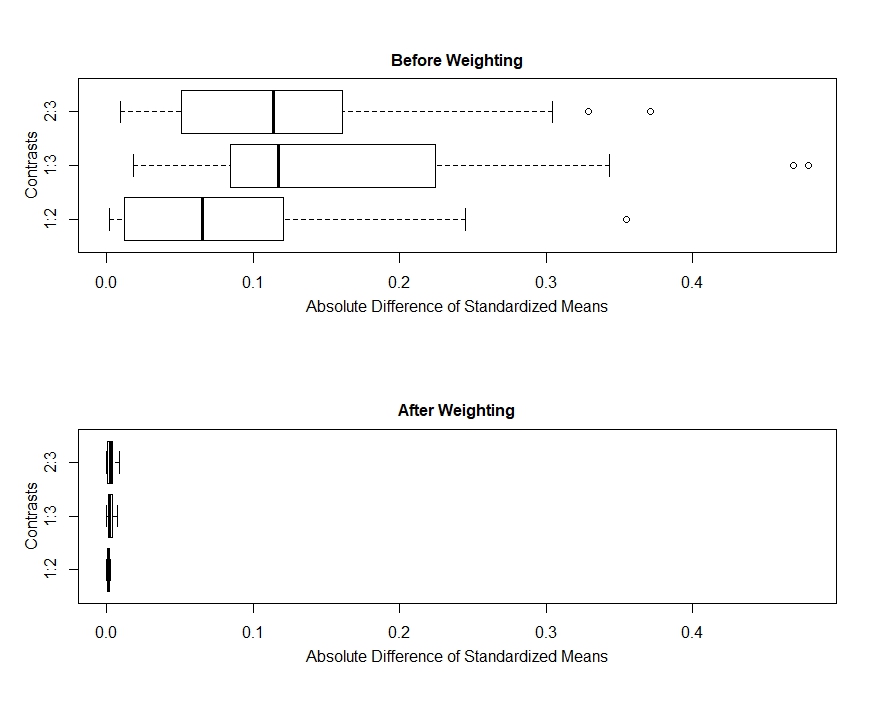


Female Black Female


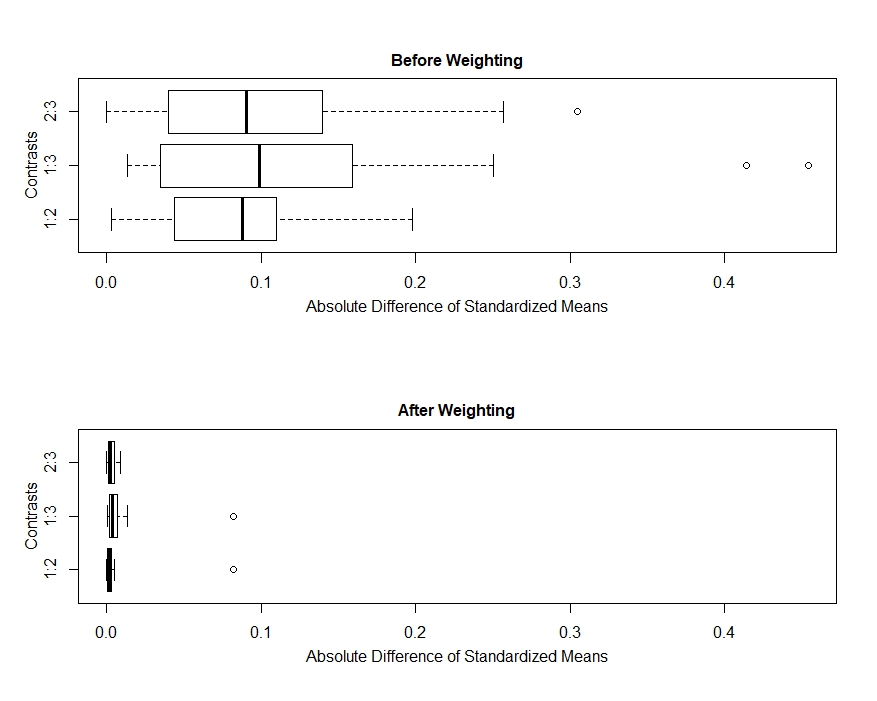


Hispanic Female

**Appendix B**. Descriptive statistics of raw variables

| Variables | Mean | SD | Min | Max |
| --- | --- | --- | --- | --- |
| **Dependent variable** |  |  |  |  |
| Reading IRT scores_W4 | 94.40 | 17.93 | 32.74 | 139.21 |
| Reading IRT scores_W6 | 111.89 | 17.22 | 45.91 | 146.42 |
| Reading IRT scores_W7 | 120.54 | 15.60 | 65.54 | 156.47 |
| Reading IRT scores_W8 | 129.00 | 14.92 | 73.40 | 155.49 |
| Reading IRT scores_W9 | 136.08 | 15.72 | 72.27 | 159.01 |
| Math IRT scores_W4 | 72.25 | 15.73 | 12.27 | 138.92 |
| Math IRT scores_W6 | 89.86 | 18.24 | 18.24 | 139.10 |
| Math IRT scores_W7 | 103.69 | 18.04 | 43.41 | 147.89 |
| Math IRT scores_W8 | 112.29 | 17.97 | 25.73 | 147.90 |
| Math IRT scores_W9 | 119.66 | 17.79 | 26.76 | 148.04 |
| **Independent variable** |  |  |  |  |
| BMI_W2 | 16.60 | 2.51 | 7.60 | 49.14 |
| **Mediating variable** |  |  |  |  |
| Approach to learning _W2 | 3.10 | 0.69 | 1.00 | 4.00 |
| Approach to learning _W4 | 3.08 | 0.70 | 1.00 | 4.00 |
| Approach to learning _W6 | 3.08 | 0.71 | 1.00 | 4.00 |
| Approach to learning _W7 | 3.08 | 0.71 | 1.00 | 4.00 |
| Approach to learning _W8 | 3.09 | 0.70 | 1.00 | 4.00 |
| Self-control_W2 | 3.18 | 0.64 | 1.00 | 4.00 |
| Self-control_W4 | 3.22 | 0.62 | 1.00 | 4.00 |
| Self-control_W6 | 3.23 | 0.63 | 1.00 | 4.00 |
| Self-control_W7 | 3.27 | 0.62 | 1.00 | 4.00 |
| Self-control_W8 | 3.28 | 0.61 | 1.00 | 4.00 |
| Interpersonal skills_W2 | 3.13 | 0.65 | 1.00 | 4.00 |
| Interpersonal skills_W4 | 3.14 | 0.66 | 1.00 | 4.00 |
| Interpersonal skills_W6 | 3.13 | 0.66 | 1.00 | 4.00 |
| Interpersonal skills_W7 | 3.13 | 0.66 | 1.00 | 4.00 |
| Interpersonal skills_W8 | 3.13 | 0.65 | 1.00 | 4.00 |
| Externalizing behaviors_W2 | 1.64 | 0.64 | 1.00 | 4.00 |
| Externalizing behaviors_W4 | 1.72 | 0.62 | 1.00 | 4.00 |
| Externalizing behaviors_W6 | 1.71 | 0.62 | 1.00 | 4.00 |
| Externalizing behaviors_W7 | 1.68 | 0.61 | 1.00 | 4.00 |
| Externalizing behaviors_W8 | 1.63 | 0.59 | 1.00 | 4.00 |
| Internalizing behaviors_W2 | 1.51 | 0.50 | 1.00 | 4.00 |
| Internalizing behaviors_W4 | 1.54 | 0.50 | 1.00 | 4.00 |
| Internalizing behaviors_W6 | 1.58 | 0.52 | 1.00 | 4.00 |
| Internalizing behaviors_W7 | 1.60 | 0.53 | 1.00 | 4.00 |
| Internalizing behaviors_W8 | 1.59 | 0.54 | 1.00 | 4.00 |
| **Covariates (at kindergarten)** |  |  |  |  |
| Sex (male=1) | 0.51 | 0.50 | 0.00 | 1.00 |
| Age (month) | 73.44 | 4.47 | 52.21 | 99.45 |
| Black | 0.13 | 0.34 | 0.00 | 1.00 |
| Hispanic | 0.25 | 0.43 | 0.00 | 1.00 |
| Asian | 0.09 | 0.28 | 0.00 | 1.00 |
| White | 0.47 | 0.50 | 0.00 | 1.00 |
| Birth weight (ounce) | 115.91 | 21.56 | 16.00 | 221.00 |
| Pre-K program participation | 0.86 | 0.34 | 0.00 | 1.00 |
| Child overall health | 3.45 | 0.78 | 0.00 | 4.00 |
| Child disability | 0.20 | 0.40 | 0.00 | 1.00 |
| Family income | 10.48 | 5.60 | 1.00 | 18.00 |
| Family size | 4.61 | 1.39 | 2.00 | 15.00 |
| Single parent | 0.29 | 0.45 | 0.00 | 1.00 |
| Parent’s educational level | 4.62 | 1.94 | 1.00 | 9.00 |
| Parent’s educational expectations | 5.23 | 1.23 | 1.00 | 7.00 |
| Home language (non-English=1) | 0.03 | 0.18 | 0.00 | 1.00 |
| Cultural participation | 2.99 | 1.68 | 0.00 | 8.00 |
| Kindergarten math scores | 49.86 | 13.34 | 11.75 | 112.54 |
| Kindergarten reading scores | 68.89 | 14.73 | 32.39 | 133.54 |
| Kindergarten science scores | 33.48 | 7.38 | 19.19 | 55.28 |
| City (residential area) | 0.34 | 0.47 | 0.00 | 1.00 |
| Suburban | 0.36 | 0.48 | 0.00 | 1.00 |
| Town | 0.08 | 0.27 | 0.00 | 1.00 |
| Rural | 0.22 | 0.42 | 0.00 | 1.00 |
| North (school locale) | 0.17 | 0.37 | 0.00 | 1.00 |
| Mid | 0.21 | 0.41 | 0.00 | 1.00 |
| South | 0.36 | 0.48 | 0.00 | 1.00 |
| West | 0.26 | 0.44 | 0.00 | 1.00 |
| School type (private =1) | 0.12 | 0.33 | 0.00 | 1.00 |
| School SES | -0.07 | 0.53 | -2.13 | 1.85 |
| School size (5 scale) | 3.44 | 1.07 | 1.00 | 5.00 |
| Percentage of school minority students | 48.97 | 34.25 | 0.00 | 100.00 |

SOURCE: U.S. Department of Education, National Center for Education Statistics, Early Childhood Longitudinal Study, Kindergarten Class of 2010–11 (ECLS-K:2011).
